# DNL-Net: deformed non-local neural network for blood vessel segmentation

**DOI:** 10.1186/s12880-022-00836-z

**Published:** 2022-06-06

**Authors:** Jiajia Ni, Jianhuang Wu, Ahmed Elazab, Jing Tong, Zhengming Chen

**Affiliations:** 1 College of Internet of Things Engineering, HoHai University, Changzhou, China; 2 Shenzhen Institutes of Advanced Technology, Chinese Academy of Sciences, Shenzhen, China; 3 School of Biomedical Engineering, Shenzhen University, Shenzhen, China; 4 Computer Science Department, Misr Higher Institute for Commerce and Computers, Mansoura, Egypt

**Keywords:** Blood vessel segmentation, Deep learning, Non-local neural network, Attention mechanisms, Spatial pyramid pooling

## Abstract

**Background:**

The non-local module has been primarily used in literature to capturing long-range dependencies. However, it suffers from prohibitive computational complexity and lacks the interactions among positions across the channels.

**Methods:**

We present a deformed non-local neural network (DNL-Net) for medical image segmentation, which has two prominent components; deformed non-local module (DNL) and multi-scale feature fusion. The former optimizes the structure of the non-local block (NL), hence, reduces the problem of excessive computation and memory usage, significantly. The latter is derived from the attention mechanisms to fuse the features of different levels and improve the ability to exchange information across channels*.* In addition, we introduce a residual squeeze and excitation pyramid pooling (RSEP) module that is like spatial pyramid pooling to effectively resample the features at different scales and improve the network receptive field.

**Results:**

The proposed method achieved 96.63% and 92.93% for Dice coefficient and mean intersection over union, respectively, on the intracranial blood vessel dataset. Also, DNL-Net attained 86.64%, 96.10%, and 98.37% for sensitivity, accuracy and area under receiver operation characteristic curve, respectively, on the DRIVE dataset.

**Conclusions:**

The overall performance of DNL-Net outperforms other current state-of-the-art vessel segmentation methods, which indicates that the proposed network is more suitable for blood vessel segmentation, and is of great clinical significance.

## Background

Many diseases result from lesions in blood vessels. For example, cerebral thrombosis is caused by blockage of blood vessels in the intracranial arteries. Therefore, the vascular segmentation is critical to the diagnosis and treatment of vascular diseases [1, 2].

With the rapid development of deep learning in the field of medical images [3–6], many deep learning model algorithms have been applied in the medical image segmentation tasks, primarily based on convolutional neural networks (CNN), have been proposed in recent years [7–14]. Due to its simple structure and excellent performance, U-Net [15] has become the backbone of many different vascular segmentation networks. DEU-Net [16] utilizes the dual encoding U-Net to capture more semantic information with multiscale convolution block. CE-Net [17], which designed a dense atrous convolution block and a residual multi-kernel pooling for further context information with multi-scale pooling operations. Although these U-Net-based architectures perform well, the weight sharing mechanism of the CNN induces these networks to extract primarily local features while ignoring global features.

To address this problem, researchers began to introduce different network structures. For example, attentional mechanism [18, 19] are introduced to capture rich contextual dependencies. Linsley et al. [20] extended the squeeze-and-excitation (SE) module [21] with a novel global-and-local attention module for visual recognition. Furthermore, spatial pyramid pooling [22, 23] and more complex backbone networks like ResNet101 [24] were introduced to improve segmentation accuracy. However, such strategies require large computation and memory resources, thus they are not very effective. Consequently, people began to use alternative strategies like long dependencies. The non-local (NL) network computes the pairwise relations between the query position and all positions to form an attention map for each query position, and can effectively extract long dependencies features. Wang et al*.* [25] combined CNN and the traditional non-local means to capture the long range dependencies in an image. Zhu et al. [26] present asymmetric non-local neural network to semantic segmentation. In the medical image segmentation task, Wang et al. [27] proposed the non-local U-Nets structure, which are equipped with flexible global aggregation blocks.

**Fig. 1:**
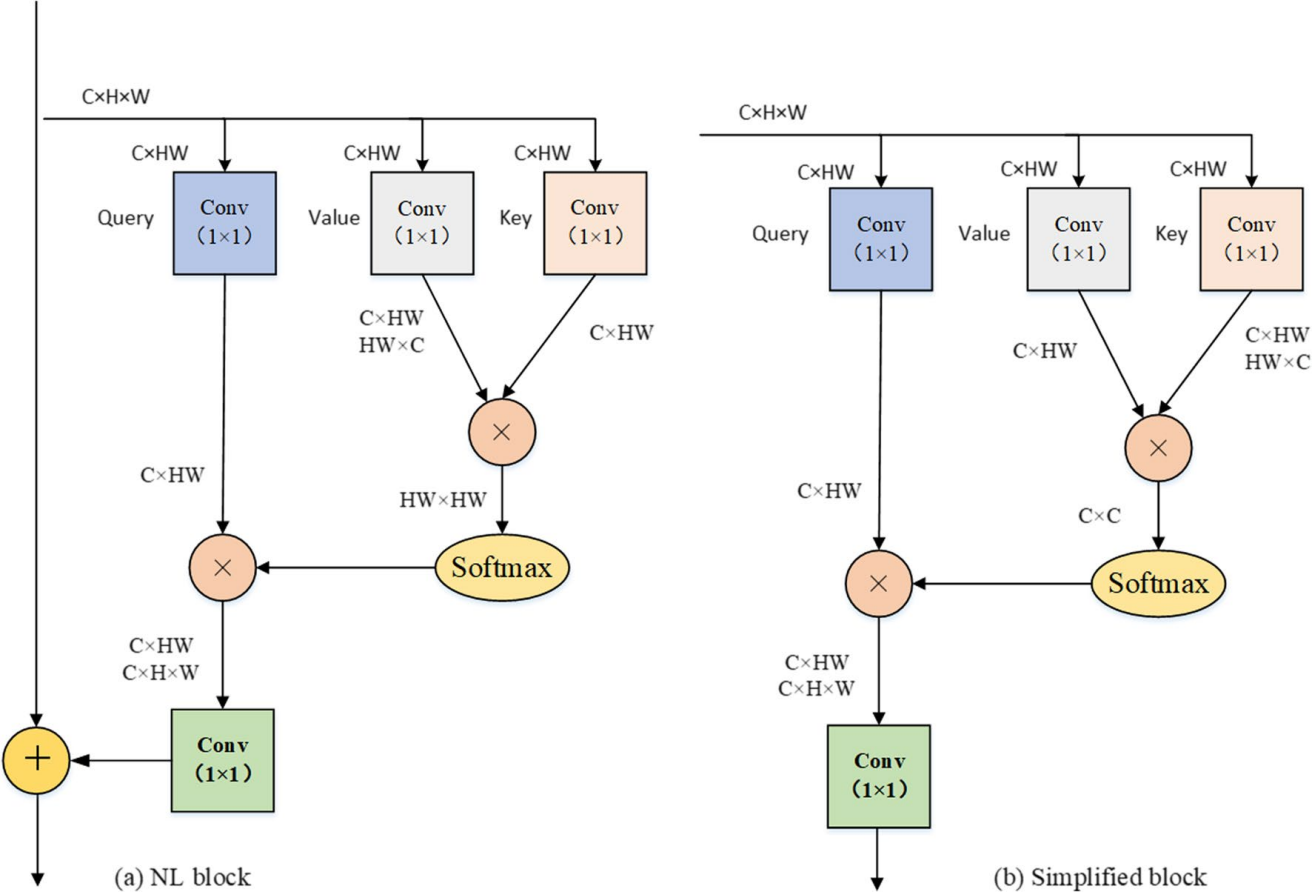
Architecture of non-local block (Embedded Gaussian) (**a**) and its simplified version (**b**). The dimensions of the input feature maps are $C \times H \times W$, ⊗ is the matrix multiplication, and ⊕ is the broadcast element-wise addition

Based on the above discussion, in the standard NL network, shown in Fig. 1a, to calculate the similarity between each location, the computational complexity is *O*(*CH*^2^*W*^2^). We observe that the complexity of NL is primarily determined by the product of the *value* and *key* branch. As long as capturing the attentional feature map in a NL network, the multiplication operation cannot be avoided. Since multiplication operation is very similar to the multiplication operation in positional attention, it is possible to have an operation that satisfies both the acquisition of an attentional feature map and the cross-channel communication of information. We know that in the attention mechanism, the calculation amount and memory occupation of channel attention are greatly reduced compared to positional attention. Thus, we can use channel attention mechanisms instead of positional attention mechanisms. In this view, the time complexity and memory occupation can be significantly decreased without sacrificing the performance.

**Fig. 2:**
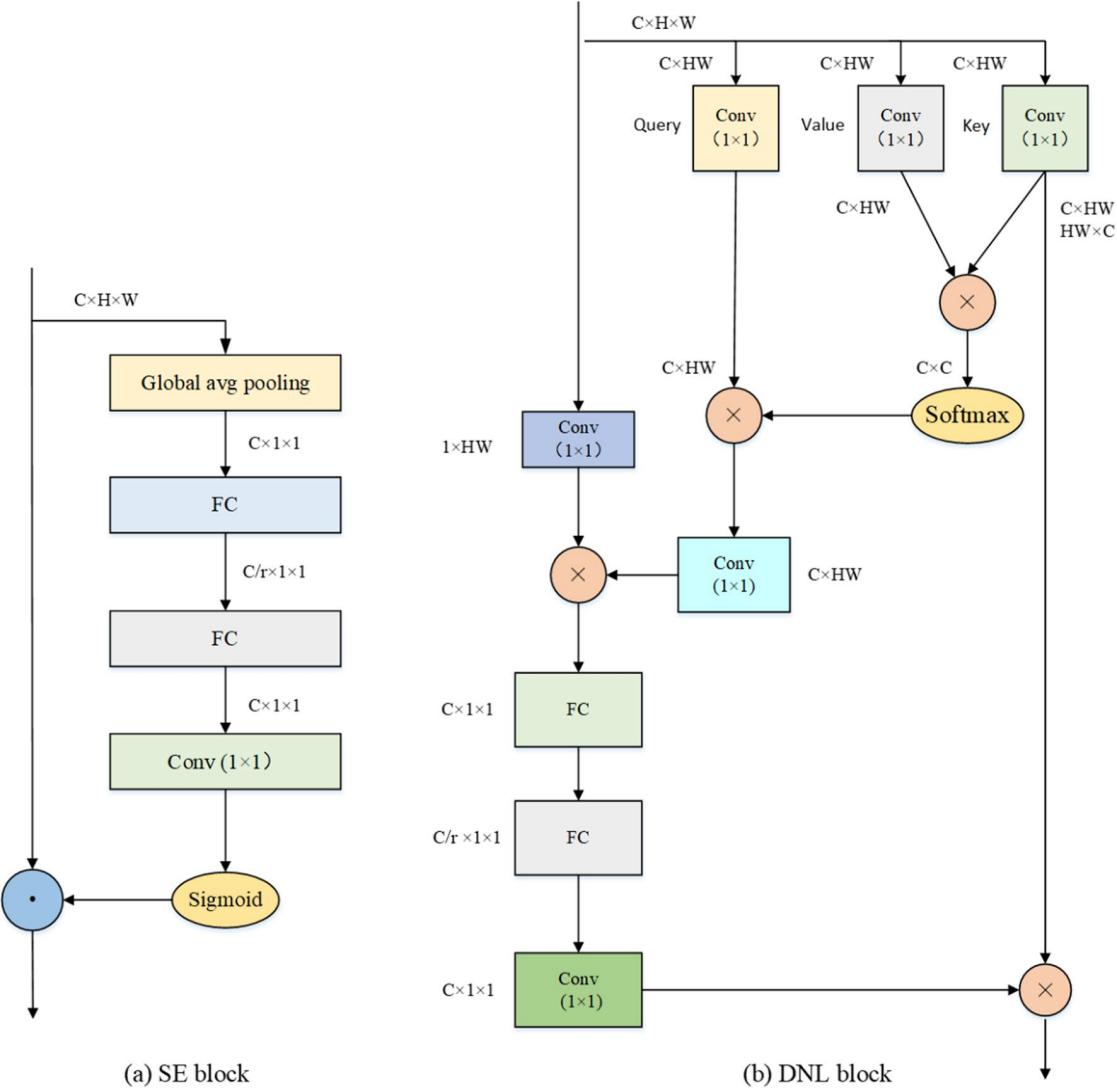
Architecture of the main blocks; (**a**) SE block and (**b**) DNL block. The dimensions of the input feature maps are $C \times H \times W$. ⊗ denotes matrix multiplication and $\odot$ denotes broadcast element-wise multiplication

The main contributions of this paper are as follows. (1) we propose a simple NL module, as shown in Fig. 1b, to reduce the complexity of the standard non-local module. Motivated by the attention mechanism strategy, we embed an SE module as shown in Fig. 2a into a simplified non-local block, which can enhance the features by aggregating them from different positions as shown in Fig. 2bWe name the new block called deformed non-local module (DNL). (2) At each stage of the decoder, we replace the vanilla skip connection of the classic U-Net model with multi-scale feature fusion (MFF) module, which can largely boost the efficiency and allow shallow features to be combined with high-level features. (3) In order to increase the receptive field of the model to adapt the variant scale of vessels by figuring out the importance of different scales, we adopt an RSEP to fuse the features of different stages of the deep network.

The rest of the paper is organized as follows: “Related work” section describes related work. “Methods” section describes our proposed segmentation method in detail. “Experiments” section discusses our experimental results. Finally, the conclusions are presented in “Conclusions” section.

## Related work

In this Section, we review the related works about semantic segmentation. Ideally, semantic segmentation methods that are based on deep learning mainly can be roughly categorized into four directions: Encoder-Decoder, Different module, Attention mechanism, and long dependencies, which are not the relationship of sequential iterations, but the relationship of parallel coexistence. They can be applied independently to semantic segmentation or applied to semantic segmentation in a combined manner.

### Encoder–decoder

The encoder-decoder structure is a classical structure in semantic segmentation, where the encoder part is used to extract features, while the decoder part is used to restore features. DeconvNet [28] used multiple deconvolutions to perform the decoding pass. Following that, U-Net [15] introduced skip-connections to connect the encoding and decoding layers, which proved quite successful in semantic segmentation of many medical image segmentation tasks. Inspired by U-Net, many semantic segmentation networks have been proposed with different coding and decoding structures [7, 29–31].

### Different module

Researchers have introduced many modules such as conditional random field (CRF) to improve segmentation accuracy of the network. For example, CNN-CRF [32] combined the fully convolutional networks (FCN) and CRF for semantic segmentation. Although this method can improve the accuracy of semantic segmentation, it has only a limited improvement. With the introduction of U-Net, various connection modules had been proposed to improve the segmentation accuracy [22, 29, 33–36]. One of the best-known methods is the DeepLab [29] which combined many different module such as atrous spatial pyramid pooling (ASPP) [37], dilated convolution [38], and encoder-decoder [28, 34].

### Attention mechanism

Inspired by the success of the SE [21], several studies adopted attention mechanisms into semantic segmentation [39–42]. The convolutional block attention module (CBAM) method [43] introduced two attention mechanisms (channel attention and spatial attention). The PARSENET [18] used the global features and a learning normalization method which improved the segmentation accuracy. Dual attention via spatial and channel attentions to capture rich contextual dependencies was also proposed in [44]. The SANet [45] introduced a novel squeeze-and-attention network architecture for obtaining an enhanced pixel-wise prediction. On the other hand, HMANet [46] used a novel attention-based framework to adaptively capture global correlations from the perspective of space, channel, and category. Also, Zhao et al. [23] proposed a pyramid feature attention network to focus on the high-level and low-level features.

### Long dependencies

Since the global features have showed great importance for semantic segmentation, researchers began to study the long dependencies features. Wang et al. [27] propose the non-local U-Nets, which are equipped with flexible global aggregation blocks, for biomedical image segmentation. Yu et al. [8] developed a network with context prior and feature aggregation to distinguish the intra-class and interclass contextual information. Also, Huang et al. [47] proposed criss-cross attention for semantic segmentation. Yue et al. [48] designed a generalized NL module that utilizes the positions of any two channels.

Different from these works, our network incorporates the channel attention and deformed non-local blocks to capture the semantic segmentation feature, while the computation time and memory are greatly reduced without compromising the segmentation accuracy.

## Methods

**Fig. 3:**
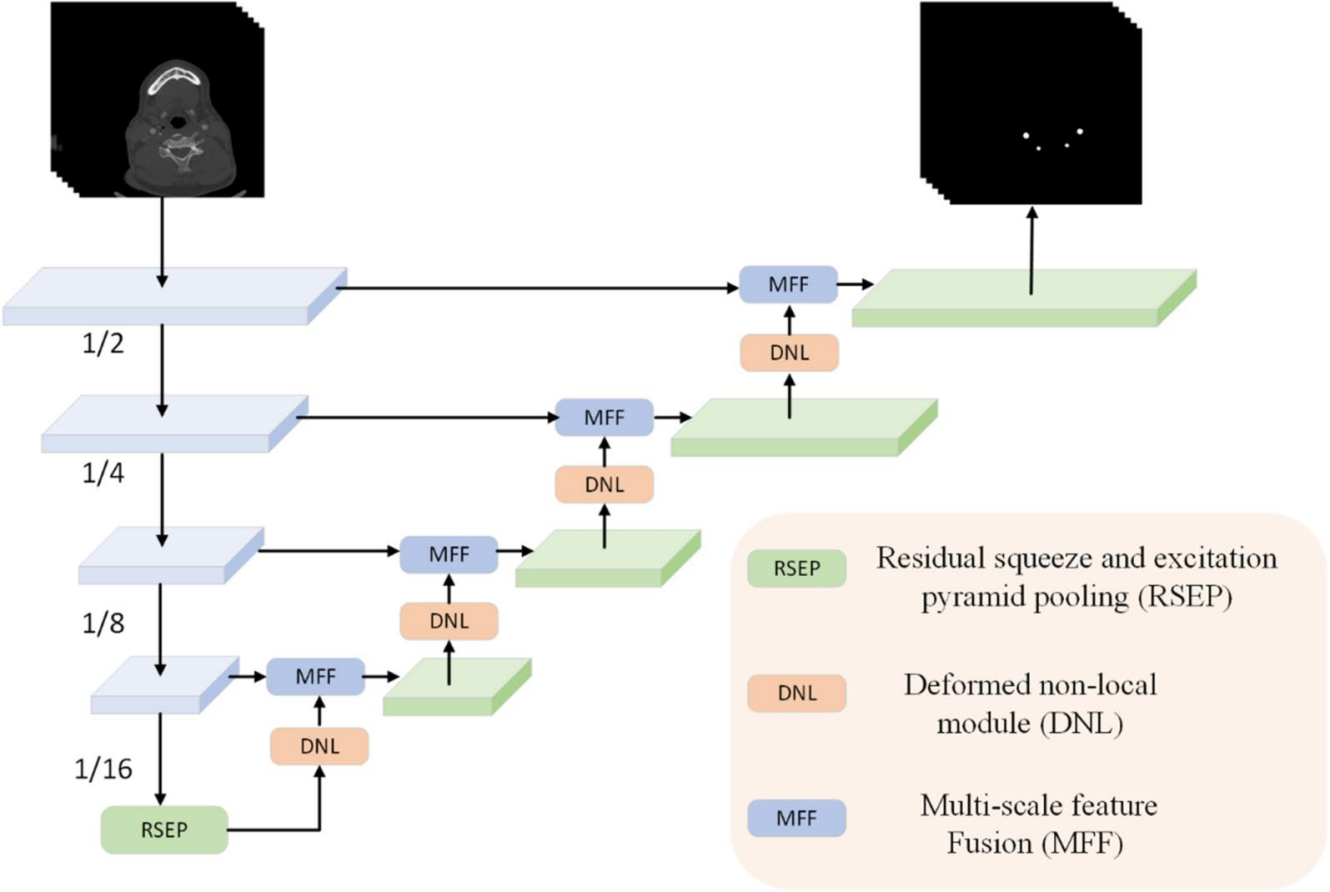
Overview of the proposed DNL-Net

**Fig. 4:**
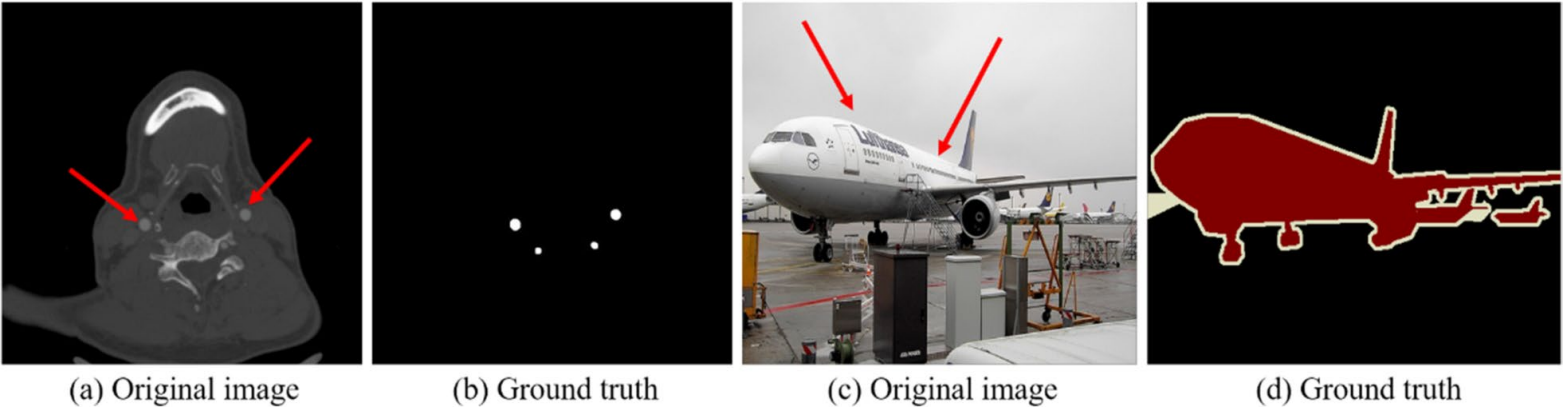
Differences between medical images and natural images

DNL-Net is a classical encoding–decoding structure network, as shown in Fig. 3. We choose the convolution and batch normalization (“Conv + BN”) to form the feature coding stage. The reason is that, medical image data usually do not contain as much information as natural images as shown in Fig. 4. In the figure, we can see that the region of interest of medical images is about 0.3% compared to the whole image, while the large region of interest of natural images is about 18.7%. When a complex network structure is used as a backbone, it is easy to lose some feature information, thereby affecting the accuracy of segmentation. We also demonstrate this conclusion in the ablation study. After feature coding stage, we use the RSEP module to increase the network's receptive field and adjust the channel information of the feature. Then, in the feature decoding phase, we use DNL and MFF to combine the shallow and high-level features.

### Non-local block

The basic NL block is shown in Fig. 1a. The way to long-range dependencies for non-locals is the self-attention mechanisms. We denote $x \in R^{H \times W \times C}$ and $y \in R^{H \times W \times C}$ as the input and output feature maps, respectively. Where, $H\;and\;W$ indicate feature map height and width and *C* indicate the feature map channel number. Mathematically, the NL block can be formulated as:

1 $$y_{i} = \frac{1}{C\left( x \right)}\mathop \sum \limits_{\forall j} f\left( {x_{i} ,x_{j} } \right)g\left( {x_{j} } \right),$$

where $i$ is the index of an output position and $j$ is the index that enumerates all possible positions. The function $f$ computes the representing relationship while function $g$ computes the representation of the input signal at the position $j$. The factor $C\left( x \right)$ indicates the normalization term.

The function $g$ can be a linear embedding such as $g\left( {x_{j} } \right) = W_{g} x_{j}$, where $W_{g}$ is a weight matrix while the function $f$ can have many different forms (embedded Gaussian, dot product, and concatenation). For example, in embedded Gaussian form the function $f$ is defined as:

2 $$f\left( {x_{i} ,x_{j} } \right) = e^{{\theta \left( {x_{i} } \right)^{T} \phi \left( {x_{j} } \right)}} ,$$

where $\theta \left( {x_{i} } \right) = W_{\theta } x_{i}$ and $\phi \left( {x_{j} } \right) = W_{\phi } \left( {x_{j} } \right)$. Similar to $W_{g}$, $W_{\theta } \;and\;W_{\phi }$ are also weight matrices. When $C\left( x \right)\;{\text{is}}\;{\text{used}}\;{\text{as}}\;\mathop \sum \limits_{\forall j} f(x_{i} ,x_{j} )$.

We note that the self-attention module is a special case of non-local operations in the embedded Gaussian version. This can be seen from the fact that for a given $i$, the $\frac{1}{C\left( x \right)}f\left( {x_{i,} x_{j} } \right)$ becomes the $softmax$ function. Finally, the NL block becomes:

3 $$y = softmax\left( {x^{T} W_{\theta }^{T} W_{\phi } x} \right)W_{g} x.$$

In addition, to simplify network computing, the dot product is generally used. Hence, the function $f$ can be defined as:

4 $$f\left( {x_{i,} x_{j} } \right) = \theta \left( {x_{i} } \right)^{T} \phi \left( {x_{j} } \right).$$

For gradient simplified calculations, the normalization term is defined as $C\left( x \right) = N$, $N$ is the number of the position in the feature map. Here, the $\theta \left( * \right) and \phi \left( * \right)$ can be implemented as1 × 1 or 1 × 1 × 1 convolutions. Finally, NL can be defined as follows:

5 $$Z_{i} = W_{z} y_{i} + x_{i} ,$$

where “ + ”operation denotes the residual connection.

### Deformed non-local (DNL)

Taking the simplest form (dot product) as an example, the most time-consuming in part NL block is the multiplication operation of function $f$ and function $g$, which has computational complexity of ${\text{O}}\left( {{\text{C}}H^{2} W^{2} } \right)$. In the semantic segmentation task, the network using the encoder-decoder module restores the feature resolution layer by layer (for example in our training phase, H × W = 512 × 512 = 262,144). Hence, the product operation of this matrix takes much time as the feature resolution becomes larger.

Therefore, in order to reduce the running time, it is necessary to reduce the complexity of the product of the weight matrix. We observe that in the NL block calculations, the function *f* cannot be avoided. Regardless of the function, basically the product of two weight matrices is needed. Therefore, the simplification operation can only occur in the step of the product of the function $f$ and the function $g$. Based on the previous discussion, we adopt the most widely-used version, *i.e.*, embedded Gaussian, as the basic NL block. In the basic NL function $f$, it mainly has three forms and it is defined as follows:

6 $$f\left( {x_{i,} x_{j} } \right) = e^{{\theta \left( {x_{i} } \right)^{T} \phi \left( {x_{j} } \right)}}$$

where $\theta \left( {x_{i} } \right) = W_{\theta } x_{i}$ and $\phi \left( {x_{j} } \right) = W_{\phi } \left( {x_{j} } \right)$. $W_{g}$, $W_{\theta } \;and\;W_{\phi }$ are weight matrices. Here, we get a matrix of $CH^{2} W^{2}$. This matrix leads to a tremendous increase in the amount of the subsequent operations. Stimulated by the channel attention network, we simplify the function $f$ as follows:

7 $$f\left( {x_{i,} x_{j} } \right) = e^{{\theta \left( {x_{i} } \right)\phi \left( {x_{j} } \right)^{T} }} .$$

In this way, we get a matrix of $C \times C$ size and the computational complexity becomes O(CHW). Thus, when the *softmax* is performed later, the operation is reduced by $\frac{HW}{C}$ times. Later, the function $f$ and the function $g$ are then subjected to a product operation to obtain a matrix of $C \times HW$ using:

8 $$y_{1} = softmax\left( {W_{\theta } xx^{T} W_{\phi }^{T} } \right)W_{g} x,$$

9 $$y_{2} = conv_{1 \times 1}^{1} \left( x \right) \times y_{1}^{T} ,$$

10 $$y_{3} = Conv_{1 \times 1}^{\frac{C}{rate}} \left( {y_{2} } \right),$$

11 $$y_{4} = Conv_{1 \times 1}^{C} \left( {y_{3} } \right)$$

12 $$y = y_{4} \times (Conv_{1 \times 1}^{T} \left( x \right))^{T} ,$$

where $Conv_{1 \times 1}^{x}$ indicates a 1 × 1 convolution with the number of channels is $x$ while C/r denotes the hidden representation dimension. “$\times$” indicates matrix multiplication operations. Different from the NL block, we add an SE module after the non-local operation, where the SE is a lightweight module and does not increase the amount of computation.

### Multi-scale feature fusion (MFF)

**Fig. 5:**
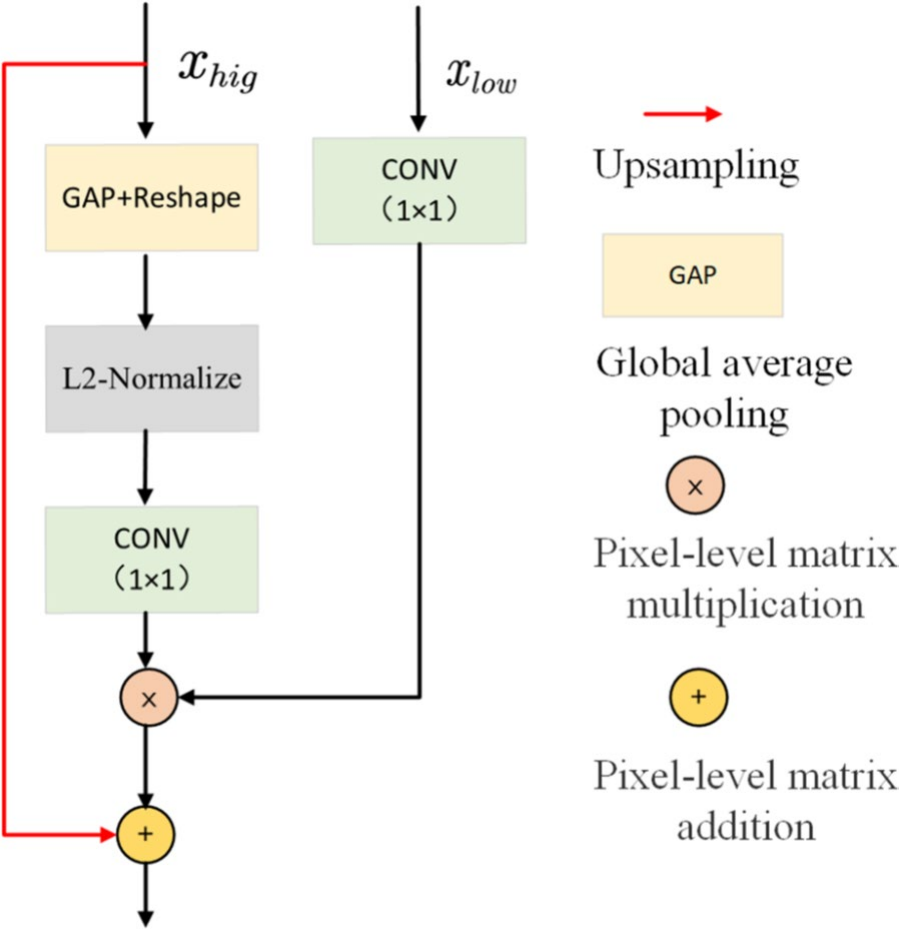
Overview of the proposed multi-scale feature fusion (MFF)

As the success of U-Net is demonstrated in different tasks, it is well-known that the feature maps connecting different levels are important for semantic segmentation. The usual connection method is addition or concatenation. The naive connection is insufficient to consider the complementariness between high-level features and low- level features. Therefore, we propose a multi-scale feature fusion (MFF) module to guide the fusion between adjacent layers based on channel attention operation, as illustrated in Fig. 5, which can be formulated as:

13 $$MFF = add\left[ {MU\left( {AV\left( {DNL\left( {x_{hig} } \right)} \right),x_{low} } \right),UP\left( {x_{hig} } \right)} \right]$$

where $MU,add$ are a pixel-level matrix multiplication and addition operation,$x_{hig}$ and $x_{low}$ represent shallow features and high-level features, respectively. $AV$ is calculated using:

14 $$AV = Conv_{1 \times 1} (Normalize\left( {GAP\left( x \right)} \right),$$

where $Conv_{1 \times 1}$ denotes the 1 × 1 convolutions, *Normalize* is the L2-Normalize operation and GAP is the global average pooling operation.

### Residual squeeze and excitation pyramid pooling (RSEP)

An essential challenging problem in semantic segmentation is that, the resolution of the image gradually decreases after multiple convolutions and pooling operations, and simultaneously, the effective receptive field gradually decreases. In this paper, we propose an RSEP technique to address this problem, which mainly relies on multiple effective receptive fields to gather at different sizes.

It is well-known that the large receptive field plays an important role in the semantic image segmentation. Generally, there are two main ways to increase the receptive field. The first is to deepen the network architecture while the second is to use the dilated convolution. In this paper, we use the structure of the spatial pyramid pooling containing a dilated convolution. The main difference from the common ASPP method is that, we combine the SE operation into the spatial pyramid pooling block. The main reason is that, the SE can re-adjust the channel information of the feature, thus, more informative features can be obtained.

**Fig. 6:**
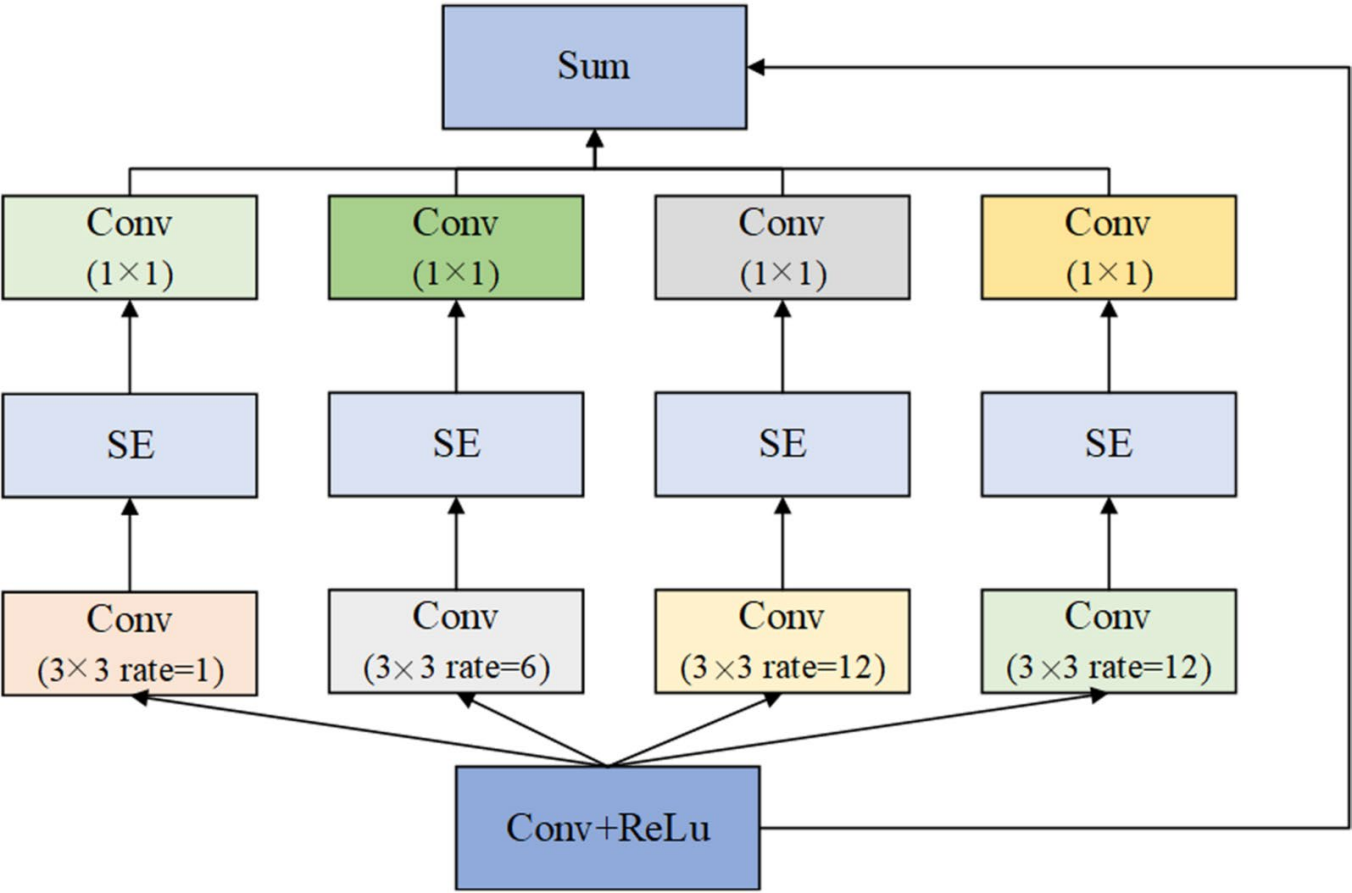
Illustrations of the RSEP module with 4 dilated convolutions

In this case, the RSEP module has four cascade branches with the gradual increment of the number of atrous convolution and SE network structure, Fig. 6. Since a large receptive field is good for acquiring much contextual information, we present 4 dilated convolutions with dilation scales being 1, 6, 12, and 12 in the RSEP. In each branch, we apply 1 × 1 convolution for rectified linear activation after every atrous convolution and SE network. Finally, we concatenate the original features with the features of the four cascaded branches feature maps.

## Experiments

### Datasets

We conduct our experiments on two different datasets: Intracranial Blood Vessel (IBV) Dataset and DRIVE Dataset. As shown in Table 1 all images are in different formats. For convenience, we convert all pictures to JPG format.

**Table 1:** An overview of the two available databases

Datasets	Quantity	Train-test split	Resolution	Format
IBV	4806	4326–480	512 × 512	.dcm
DRIVE	40	20–20	565 × 584	.tiff

The total number of images, the training and test split, the image size (width × height)

#### Intracranial blood vessel (IBV) dataset

The intracranial blood vessel dataset in this study is a self-collected dataset from a local hospital in Shenzhen, China. The imaging modality of this dataset is computed tomography angiography (CTA). There are 4326 CTA images (20 patients) of intracranial blood vessels with dimension 512 × 512 in the original dataset for training. In addition, we used two new patients (480 CTA images) that were not included in the training and validation sets as the testing set. We further augmented the training dataset to increase the number of samples to avoid the potential risk of overfitting. Specifically, we performed data augmentation in three ways, *i.e*., affine transformation, rotation, and vertical flip operations. Each image was contrast enhanced before data augmentation. During the training process, 20% of training images were used as validation set, while the remainder 80% as a training set.

#### The DRIVE dataset

**Fig. 7:**
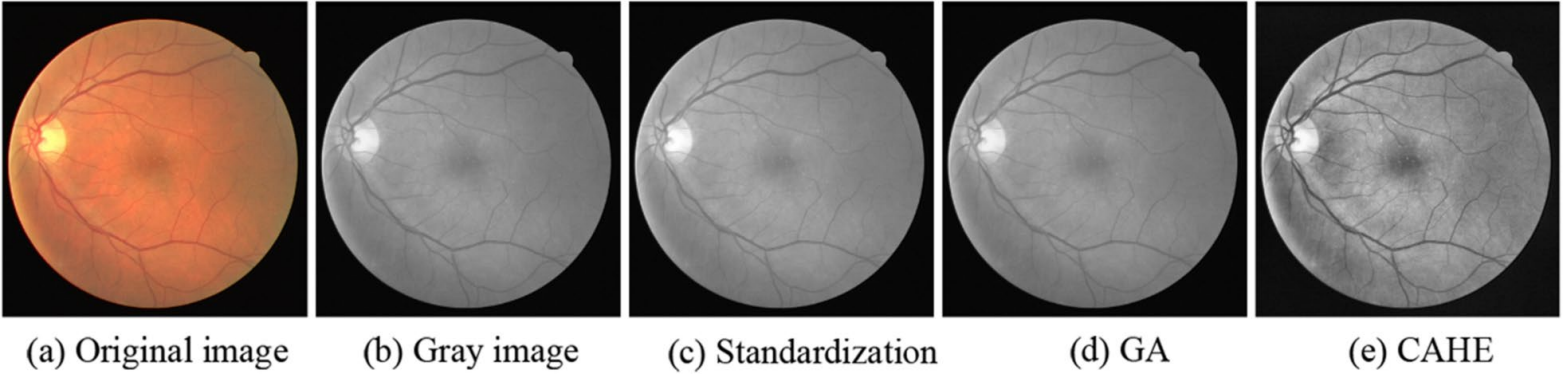
Data augmentation. We apply four strategies for data augmentation, including gray-scale conversion, standardization, gamma adjustment (GA), and contrast-limited adaptive histogram equalization (CAHE)

The images of the DRIVE dataset were obtained from a diabetic retinopathy screening program in the Netherlands. The screening population consisted of 400 diabetic subjects between 25 and 90 years of age, the size of each original image is 565 × 584 pixels. A set of 40 images were randomly selected. In the data set, these pictures were divided into 20 images for training and 20 for testing, in order to make a fair comparison with other algorithms, we also adopted this division method. In this experiment, we performed data augmentation in four ways, including gray-scale conversion, standardization, contrast-limited adaptive histogram equalization (CAHE), and gamma adjustment (GA) as shown in Fig. 7. In addition, we used image patches for training. Specifically, each 96 × 96 patch was obtained by randomly selecting its center inside the full image.

### Implementation details

Our implementation is based on the Keras deep learning library [49] with TensorFlow [50] as backend running on an Ubuntu 16.04 system with CPU Intel Core i7-5960X, GPU NVIDIA GeForce Titan XP, and 24 GB of RAM.We use the ADAM [51] optimizer with β1 = 0.5, and β2 = 0.999 and initial learning rate $lr$ = 1e–3. The $lr$ is updated during the training process by multiplying it by ${ }\left( {1 - \left( {\frac{epoch - 1}{{totalepoch}}} \right)^{power} } \right)$, where *power* is set to 0.9. The maximum number of epochs is set to 200. For DRIVE, we randomly crop out the resolution patches 96 × 96 from the original images as the inputs for training. In addition, in this paper, the loss function is based on combination of the Dice loss and the weighted cross-entropy loss as suggested in [52].

To quantitatively evaluate the proposed method, we adopt the Dice coefficient (Dice), the Mean IoU (mean of class-wise intersection over union) on the intracranial blood vessel dataset. The Dice is defined as follows:

15 $$Dice = \frac{{2 \mathop \sum \nolimits_{i}^{N} p\left( {k,i} \right)q\left( {k,i} \right)}}{{\mathop \sum \nolimits_{i}^{N} p^{2} \left( {k,i} \right) + \mathop \sum \nolimits_{i}^{N} q^{2} \left( {k,i} \right)}},$$

where N is the number of pixels*,* $p\left( {k,i} \right) \in \left[ {0,1} \right]$, and ${\text{q}}\left( {{\text{k}},{\text{i}}} \right) \in \left[ {0,1} \right]$ are, respectively, the predicted probability and ground truth labels for class k*.* On the other hand, the mean *IoU* is calculated using:

16 $$Mean IoU = \frac{1}{k}\mathop \sum \limits_{i = 0}^{k} \frac{{p_{ii} }}{{\mathop \sum \nolimits_{j = 0}^{k} p_{ij} + \mathop \sum \nolimits_{j = 0}^{k} p_{ji} - p_{ii} }}$$

where $k$ represents total number of classes,${ }p_{ij}$ are pixels whose real pixel class is $i$ are predicted as the total number of classes $j$, and $p_{ii}$ are pixels whose real pixel class is $i$ are predicted as the total number of classes $i$.

For quantitatively analyze the proposed method on the DRIVE dataset, several important metrics are utilized, including sensitivity (SE), specificity (SP) and accuracy (ACC), which are calculated by the following equations:

17 $$SE = \frac{{\left| {TP} \right|}}{{\left| {TP + FN} \right|}}$$

18 $$SP = \frac{{\left| {TN} \right|}}{{\left| {FP + TN} \right|}}$$

19 $$ACC = \frac{{\left| {TP + TN} \right|}}{{\left| {TP + TN + FN + FP} \right|}}$$

where *TP* and *FP* are the variables of true positive and false positive, which represent the number of blood vessel pixels correctly segmented and the number of background pixels that are incorrectly segmented by the model, respectively. Correspondingly, *TN* is the variable of true negative, which represents the number of background pixels that correctly segmented. *FN* is the variable of false negative, which represents the blood vessel pixel that is incorrectly marked as a background pixel. Additionally, the area under curve (AUC) of receiver operating characteristic curve (ROC) is also employed, which are based on the recall and precision to measure the segmentation performance.

### Performance evaluation

#### Performance on the intracranial blood vessel dataset

Though our DNL-Net is based on 2D CT slice images, the 3D surface is reconstructed for intuitive visualization of the segmented vasculatures, it can be seen that there are some noises on the surface as isolated objects, arising from the misclassifications. Since the entire intracranial arterial vasculature is a 3D topology, there will not be a single unconnected vessel as the mis-segmented noise is not connected to the entire blood vessel. Therefore, we remove some areas or noises, accounting for less than 0.03% of the entire blood vessel.

**Fig. 8:**
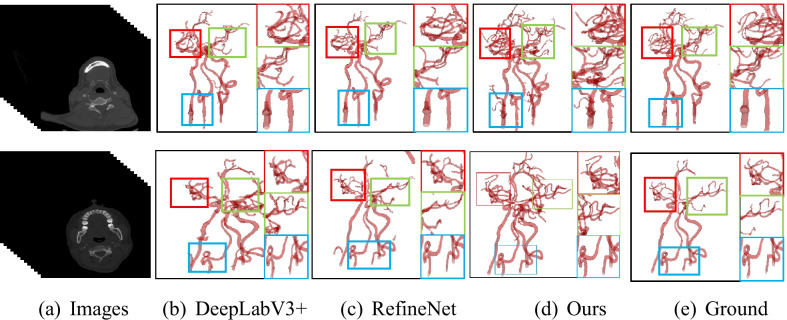
Qualitative comparisons with DeepLab-V3 + and RefineNet. The red, green and blue rectangles mark where our method is particularly superior to the others

Results of other state-of-the-art semantic segmentation solutions to intracranial blood vessel data are summarized in Table 2. These results were obtained under the same experimental conditions and the same data pretreatment. The Dice coefficient of segmentation accuracy increased from 76.14 to 96.63%, and the accuracy of Mean IoU increased from 66.53 to 92.93%. In particular, as we can see in Fig. 8, DNL-Net has more details than other methods. The main reason for this is that, the DNL, MFF the RSEP modules can well-preserve the information of medical images.

**Table 2:** Comparisons of the proposed methods against state-of-the-art methods on the intracranial blood vessel dataset

Method	Dice coefficient (%)	Mean IoU (%)
U-Net	87.32	86.48
SegNet	88.40	81.63
FCN16s	76.14	66.53
DenseASPP	84.38	81.80
Deeplab V3 +	90.70	87.83
RefineNet	91.68	76.72
ENet	85.97	81.72
BiSeNet	92.92	89.33
SA-Net	95.89	91.58
SSCA-Net	96.20	92.70
DNL-Net	96.63	92.93

#### Performance on the DRIVE dataset

Here, we report the segmentation results on the DRIVE dataset. To compare the performance of the proposed method on vessel detection, we adopt the *Sen* and *ACC* metrics, respectively. We also adopt the area under receiver operation characteristic curve (AUC) to measure segmentation performance.

**Fig. 9:**
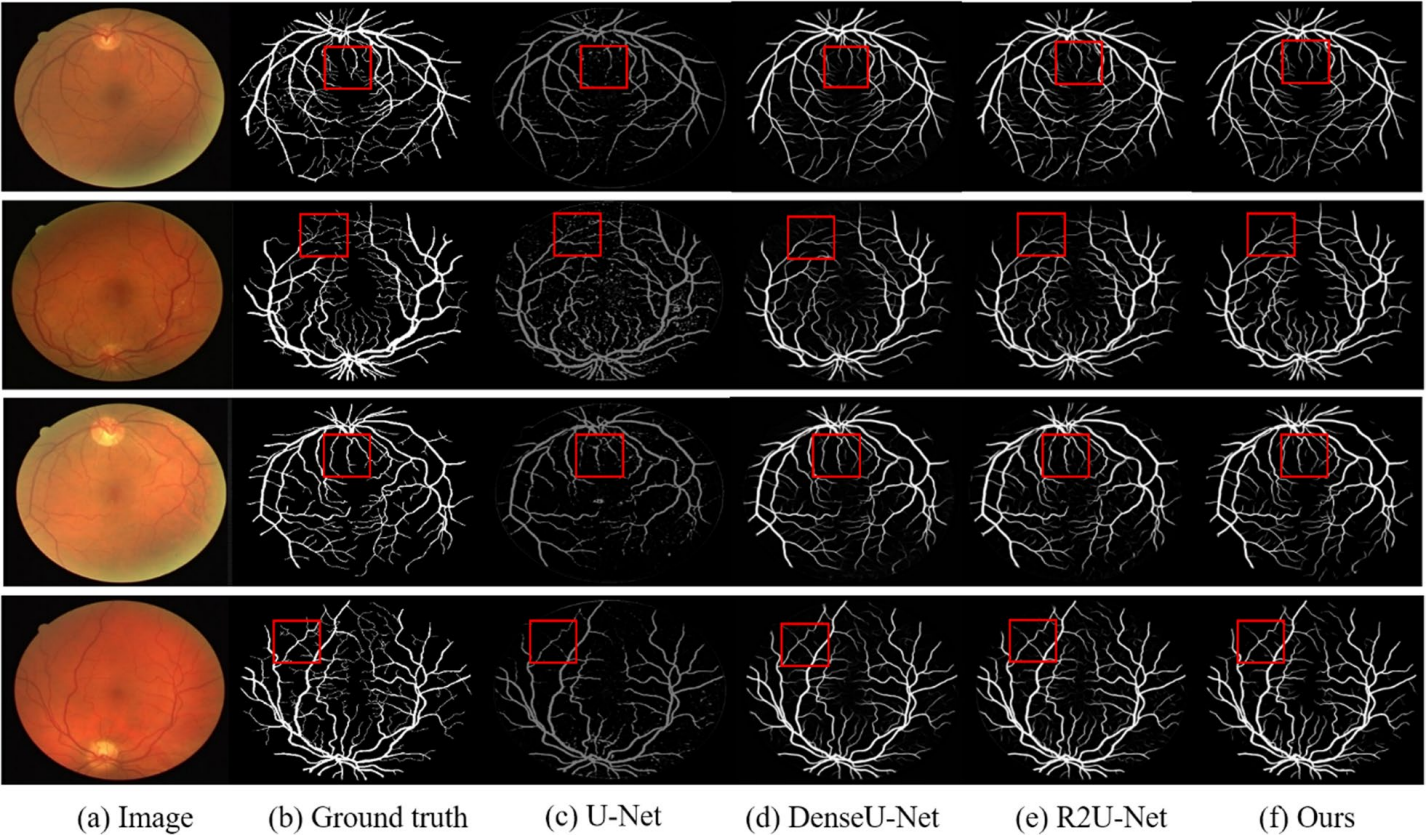
Qualitative comparisons of the proposed DNL-Net、U-Net、DenseU-Net and R2U-Net on the DRIVE database

Similarly, we compared the proposed DNL-Net with the state-of-the-art methods. The results summarized in Table 3 clearly demonstrate the superior segmentation improvements achieved by our method compared with competing methods. Note that, all baseline models are obtained directly from results provided by the authors. In this table, the proposed DNL-Net achieved 98.37%, 86.64%, and 96.10% for AUC, Sen, and Acc metrics, respectively, which are better than other methods. It can also be seen that, the AUC increased from 86.20 to 98.37% and Sen score increased from 72.50 to 86.64% while the Acc increased from 94.42 to 96.10%. Some examples for visual comparison are shown in Fig. 9.

**Table 3:** Performance comparisons of the proposed method against state-of-the-art methods on DRIVE dataset using different performance metrics

Method	Sen (%)	Acc (%)	AUC (%)
Azzopadi et al. [53]	76.55	94.42	96.14
Roychowdhury et al. [54]	72.50	95.20	96.72
Zhao et al. [55]	74.20	95.40	86.20
U-Net [15]	73.44	95.23	97.44
DeepVessel [56]	76.03	95.23	97.52
Li et al. [57]	75.69	95.27	97.38
Melinscak et al. [58]	–	94.66	97.49
DEU-Net [16]	79.40	95.67	97.72
CE-Net [17]	83.09	95.45	97.79
DenseU-Net [7]	80.40	96.04	97.97
R2U-Net [36]	83.18	95.93	98.11
SA-Net [42]	82.52	95.69	98.22
SSCA-Net [35]	83.52	96.14	98.20
DNL-Net	86.64	96.10	98.37

#### Computation efficiency

As discussed in “Deformed non-local (DNL)”, the DNL is much more efficient than the standard NL block. We hereby give a quantitative comparison between our DNL and the standard NL blocks in the following aspects: number of parameters and GPU computation time (*ms*). In our network, the NL and DNL blocks receive two different patch sizes (96 × 96 and 192 × 192). For fair comparison, the testing environment is kept identical for these two blocks, that is, two Titan Xp GPU under CUDA 9.0 without other ongoing programs.

We compare the GPU times of DNL and a standard NL, averaging the running time of every epoch. In the subsection of Deformed Non-Local (DNL), we known that DNL is roughly $\frac{HW}{C}$ times less in matrix computation, when the H and W the larger the difference between the two performance is greater, so when the image resolution of 96 × 96, the difference is not obvious, and to 192 × 192 can see the difference. As can be seen from the Table 4, In terms of time, DNL is also 2.5 times faster than a non-local block for a 192 × 192 input on DRIVE dataset.

**Table 4:** Parameters and GPU computation time (s/epoch) comparisons between the NL block and our DNL

Method	Input size	Time (ms/epoch)
NL	96 × 96	40
DNL	96 × 96	41
NL	192 × 192	154
DNL	192 × 192	48

Batch size is 4. The lower values indicate better performance

### Ablation study

In this Section, we performed extensive experiments to verify the efficacy of the proposed method. We also gave several design choices to show their influences on the results. Here *Baseline* is our redesigned U-shaped network structure, using the same coding and decoding layers and loss functions of DNL-Net. Therefore, the accuracy is significantly improved compared to the original U-Net.

#### Efficacy of the DNL, MFF and RSEP

We designed some experiments to verify the effectiveness of the two proposed modules. In addition, we also used the MFF module. However, we did not independently verify the effectiveness of MFF for semantic segmentation Since the MFF module is immediately following the DNL in the DNL-Net, we verify the DNL and MFF as one big module in our verification. By adding the RSEP to the *Baseline* model, the score of Dic is improved from 93.72 to 94.46%, and the score of Mean IoU is improved from 90.29 to 91.96%, as shown in Table 5. In addition, we added the DNL + MFF structure to the *Baseline* model, the score of Dic coefficient is improved from 93.72 to 95.76%. the sore of Mean IoU is improved from 90.29 to 91.92%. and added the ASPP with *Baseline* + *DNL* + *MFF*, the score of Dic is slightly improved from 95.76 to 96.01%. the sore of Mean IoU is slightly improved from 91.92 to 92.93%. Finally, we replace the ASPP with RSEP and find that the segmentation accuracy is also improved.

**Table 5:** Performance comparisons of context aggregation approaches on the intracranial blood vessel dataset

Method	Dice coefficient (%)	Mean IoU (%*)*
Baseline	93.72	90.29
Baseline + RSEP	94.46	91.96
Baseline + ASPP	94.17	91.97
Baseline + DNL + MFF	95.76	91.92
Baseline + DNL + MFF + ASPP	96.01	92.42
DNL-Net	96.63	92.93

#### Analysis on pretrained networks

**Fig. 10:**
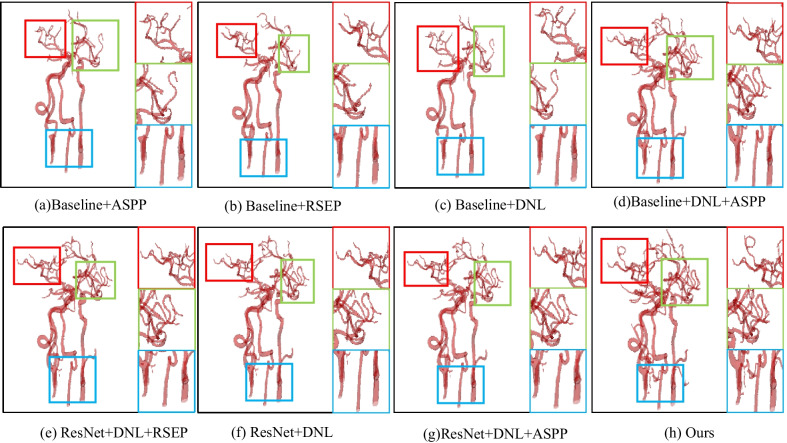
Segmentation results of the intracranial artery dataset. Our DNL-Net can effectively segment intracranial arteries while preserving more image details

Recent work [59] points out that ImageNet pre-training is no better than the original feature encoder in terms of model training accuracy. We do ablation learning on Intracranial blood vessel data sets because the data sets contain a large amount of data, which can better verify the potential of the network. On the intracranial arterial blood vessel dataset, comparing with DNL-Net, we can see that *ResNet50* + *DNL* + *MFF* + *RSEP* has increased from 94.72 to 96.63% in Dic and Mean IoU increased from 90.65 to 92.93%. The results in Fig. 10 and Table 6 have demonstrated the effectiveness of without pre-training weights is no worse than using weights.

**Table 6:** Effect of the proposed modules with the pretrained networks on the intracranial blood vessel dataset

Method	Dice coefficient (%)	Mean IoU (%)
ResNet50 + DNL + MFF	94.72	90.65
ResNet50 + DNL + MFF + ASPP	94.30	90.32
ResNet50 + DNL + MFF + RSEP	93.83	88.99
DNL-Net	96.63	92.93

### Limitations

Currently, we have used 2D images for semantic segmentation, but 3D graphics are usually dominant in medical graphics. However, 3D images contain more information than 2D images, which commonly happens in medical imaging tasks. Therefore, we should pay more attention on the 3D images. Moreover, since we were also unable to obtain other more effective cerebrovascular datasets to validate the robustness of the proposed method. We subsequently applied the DNL-net method to different medical image segmentation tasks.

To demonstrate the advantage of the proposed method in detecting very fine vessels, we further employed fundus vessels data to validate the network's segmentation ability for fine vessels. The proposed DNL-Net is capable of effectively capturing multi-scale contextual information and promoting the fusion of the features at different levels to obtain more semantic representations. The statistical results of comparisons among the proposed network and other state-of-the-art methods on the DRIVE dataset are as shown in Table 4, which clearly show the superiority of the proposed method in achieving better segmentation performance for the thin vessels.

## Conclusions

This paper presented a novel deep network model for medical image segmentation. Our approach mainly used two attention mechanisms to analyze the context information of the entire network. To obtain global contextual information, we introduced a DNL and MFF module to obtain the feature information of the image. In addition, an RSEP module was devised to increase the size of the receptive field of the network while learning more features. Finally, we adopted a weighted cross-entropy loss function to improve the effectiveness of the training process. Moreover, it was demonstrated that, the proposed DNL module had a very good speed improvement over the original NL module. At the same time, the memory usage was greatly reduced. Furthermore, we also tested the feature encoder module instead of the ResNet50 pre-training model which greatly reduced the training time and tackled the problem of network overfitting as well. We tested the proposed method on 2 medical image datasets and performed extensive comparisons against various state-of-the-art methods. Our method attained better performance in terms of Dice, Mean IoU, Sen, Acc, and AUC metrics as well as high speed and low memory consumption.

## Data Availability

The dataset used and/or analyzed in this study are available from the corresponding author upon reasonable request.

## References

[1] Carmeliet P, Jain RK (2000). Angiogenesis in cancer and other diseases. Nature.

[2] Campochiaro PA (2015). Molecular pathogenesis of retinal and choroidal vascular diseases. Prog Retin Eye Res.

[3] Chaudhuri S, Chatterjee S, Katz N, Nelson M, Goldbaum M (1989). Detection of blood vessels in retinal images using two-dimensional matched filters. IEEE Trans Med Imaging.

[4] Chen Y, Li D, Zhang X, Jin J, Shen Yi (2021). Computer aided diagnosis of thyroid nodules based on the devised small-datasets multi-view ensemble learning. Med Image Anal.

[5] Ge R, Yang G, Chen Y, Luo L, Feng C, Ma H, Ren J, Li S (2019). K-net: integrate left ventricle segmentation and direct quantification of paired echo sequence. IEEE Trans Med Imaging.

[6] Luo L, Lequan Yu, Chen H, Liu Q, Wang Xi, Jiaqi Xu, Heng P-A (2020). Deep mining external imperfect data for chest X-ray disease screening. IEEE Trans Med Imaging.

[7] Guan S, Khan AA, Sikdar S, Chitnis PV (2019). Fully dense UNet for 2-D sparse photoacoustic tomography artifact removal. IEEE J Biomed Health Inform.

[8] Yu C, Wang J, Gao C, Yu G, Shen C, Sang N. Context prior for scene segmentation. In: Proceedings of the IEEE/CVF conference on computer vision and pattern recognition. 2020.

[9] Zheng S, Cornelissen LJ, Cui X, Jing X, Veldhuis RNJ, Oudkerk M, van Ooijen P. Efficient convolutional neural networks for multi-planar lung nodule detection: improvement on small nodule identification. arXiv e-prints. http://arxiv.org/abs/2001.04537 (2020). 10.1002/mp.14648 PMC7986069 33300162

[10] Li L, Verma M, Nakashima Y, Nagahara H, Kawasaki R. Iternet: retinal image segmentation utilizing structural redundancy in vessel networks. In: Proceedings of the IEEE/CVF winter conference on applications of computer vision. 2020.

[11] Shi Z, Wang T, Huang Z, Xie F, Liu Z, Wang B, Xu J (2021). MD-Net: a multi-scale dense network for retinal vessel segmentation. Biomed Signal Process Control.

[12] Yicheng Wu, Xia Y, Song Y, Zhang Y, Cai W (2020). NFN+: a novel network followed network for retinal vessel segmentation. Neural Netw.

[13] Khan A, Sohail A, Zahoora U, Qureshi AS (2020). A survey of the recent architectures of deep convolutional neural networks. Artif Intell Rev.

[14] Agn M, af Rosenschöld PM, Puonti O, Lundemann MJ, Mancini L, Papadaki A, Thust S, Ashburner J, Law I, Van Leemput K (2019). A modality-adaptive method for segmenting brain tumors and organs-at-risk in radiation therapy planning. Med Image Anal.

[15] Ronneberger O, Fischer P, Brox T. U-net: convolutional networks for biomedical image segmentation. In: International conference on medical image computing and computer-assisted intervention. 2015.

[16] Wang B, Qiu S, He H. Dual encoding u-net for retinal vessel segmentation. In: International conference on medical image computing and computer-assisted intervention. 2019.

[17] Gu Z, Cheng J, Fu H, Zhou K, Hao H, Zhao Y, Zhang T, Gao S, Liu J (2019). CE-Net: context encoder network for 2D medical image segmentation. IEEE Trans Med Imaging.

[18] Liu W, Rabinovich A, Berg AC. Parsenet: looking wider to see better. arXiv preprint http://arxiv.org/abs/1506.04579 (2015).

[19] Fan T, Wang G, Li Y, Wang H (2020). Ma-net: a multi-scale attention network for liver and tumor segmentation. IEEE Access.

[20] Linsley D, Shiebler D, Eberhardt S, Serre T. Global-and-local attention networks for visual recognition. arXiv preprint http://arxiv.org/abs/1805.08819 (2018).

[21] Hu J, Shen L, Sun G. Squeeze-and-excitation networks. 7. arXiv preprint http://arxiv.org/abs/1709.01507. (2017). 10.1109/TPAMI.2019.2913372 31034408

[22] Zhao H, Shi J, Qi X, Wang X, Jia J. Pyramid scene parsing network. In: IEEE conference on computer vision and pattern recognition (CVPR). 2017.

[23] Li H, Xiong P, An J, Wang L. Pyramid attention network for semantic segmentation. arXiv preprint http://arxiv.org/abs/1805.10180 (2018).

[24] He K, Zhang X, Ren S, Sun J. Deep residual learning for image recognition. In: Proceedings of the IEEE conference on computer vision and pattern recognition. 2016.

[25] Wang X, Girshick R, Gupta A, He K. Non-local neural networks. In: Proceedings of the IEEE conference on computer vision and pattern recognition. 2018.

[26] Zhu Z, Xu M, Bai S, Huang T, Bai X. Asymmetric non-local neural networks for semantic segmentation. In: Proceedings of the IEEE international conference on computer vision. 2019.

[27] Wang Z, Zou N, Shen D, Ji S. Non-local u-nets for biomedical image segmentation. In: Proceedings of the AAAI conference on artificial intelligence. 2020.

[28] Noh H, Hong S, Han B. Learning deconvolution network for semantic segmentation. In: Proceedings of the IEEE international conference on computer vision. 2015.

[29] Chen LC, Papandreou G, Kokkinos I, Murphy K, Yuille AL (2018). DeepLab: semantic image segmentation with deep convolutional nets, atrous convolution, and fully connected CRFs. IEEE Trans Pattern Anal Mach Intell.

[30] Norman B, Pedoia V, Majumdar S (2018). Use of 2D U-Net convolutional neural networks for automated cartilage and meniscus segmentation of knee MR imaging data to determine relaxometry and morphometry. Radiology.

[31] Livne M, Rieger J, Aydin OU, Taha AA, Akay EM, Kossen T, Sobesky J, Kelleher JD, Hildebrand K, Frey D (2019). A U-Net deep learning framework for high performance vessel segmentation in patients with cerebrovascular disease. Front Neurosci.

[32] Alansary A, Kamnitsas K, Davidson A, Khlebnikov R, Rajchl M, Malamateniou C, Rutherford M, Hajnal JV, Glocker B, Rueckert D. Fast fully automatic segmentation of the human placenta from motion corrupted MRI. In: International conference on medical image computing and computer-assisted intervention. 2016.

[33] Yang M, Yu K, Zhang C, Li Z, Yang K. DenseASPP for semantic segmentation in street scenes. In: Proceedings of the IEEE conference on computer vision and pattern recognition. 2018.

[34] Chen L-C, Zhu Y, Papandreou G, Schroff F, Adam H. Encoder-decoder with atrous separable convolution for semantic image segmentation. arXiv preprint http://arxiv.org/abs/1802.02611 (2018).

[35] Ni J, Wu J, Tong J, Wei M, Chen Z. SSCA-Net: simultaneous self-and channel-attention neural network for multi-scale structure-preserving vessel segmentation. 2020. 10.1155/2021/6622253 PMC8026298 33860043

[36] Alom MZ, Hasan M, Yakopcic C, Taha TM, Asari VK (2019). Recurrent residual U-Net for medical image segmentation. J Med Imaging.

[37] Chen L-C, Papandreou G, Kokkinos I, Murphy K, Yuille AL (2017). Deeplab: semantic image segmentation with deep convolutional nets, atrous convolution, and fully connected crfs. IEEE Trans Pattern Anal Mach Intell.

[38] Yu F, Koltun V. Multi-scale context aggregation by dilated convolutions. arXiv preprint http://arxiv.org/abs/1511.07122 (2015).

[39] Li Y, Yang J, Ni J, Elazab A, Jianhuang Wu (2021). TA-Net: triple attention network for medical image segmentation. Comput Biol Med.

[40] Guo C, Szemenyei M, Yi Y, Wang W, Chen B, Fan C. Sa-unet: spatial attention u-net for retinal vessel segmentation. In: 2020 25th International conference on pattern recognition (ICPR). 2021.

[41] Zhang C, Jingben Lu, Hua Q, Li C, Wang P (2022). SAA-Net: U-shaped network with Scale-Axis-Attention for liver tumor segmentation. Biomed Signal Process Control.

[42] Jingfei Hu, Wang H, Wang J, Wang Y, He F, Zhang J (2021). SA-Net: A scale-attention network for medical image segmentation. PLoS ONE.

[43] Woo S, Park J, Lee J-Y, Kweon IS. Cbam: convolutional block attention module. In: Proceedings of the European conference on computer vision (ECCV). 2018.

[44] Fu J, Liu J, Tian H, Li Y, Bao Y, Fang Z, Lu H. Dual attention network for scene segmentation. In: Proceedings of the IEEE conference on computer vision and pattern recognition. 2019.

[45] Zhong Z, Lin ZQ, Bidart R, Hu X, Daya IB, Li Z, Zheng W-S, Li J, Wong A. Squeeze-and-attention networks for semantic segmentation. In: Proceedings of the IEEE/CVF conference on computer vision and pattern recognition. 2020.

[46] Niu R. Hmanet: hybrid multiple attention network for semantic segmentation in aerial images. arXiv preprint http://arxiv.org/abs/2001.02870 (2020).

[47] Huang Z, Wang X, Huang L, Huang C, Wei Y, Liu W. Ccnet: criss-cross attention for semantic segmentation. arXiv preprint http://arxiv.org/abs/1811.11721 (2018). 10.1109/TPAMI.2020.3007032 32750802

[48] Yue K, Sun M, Yuan Y, Zhou F, Ding E, Xu F. Compact generalized non-local network. I: Advances in neural information processing systems. 2018.

[49] Chollet F. Keras. 2015.

[50] Abadi M, Agarwal A, Barham P, Brevdo E, Chen Z, Citro C, Corrado GS, Davis A, Dean J, Devin M. TensorFlow: large-scale machine learning on heterogeneous systems, 2015. Software available from tensorflow. org. 2015;1(2).

[51] Kingma DP, Ba J. Adam: a method for stochastic optimization. arXiv preprint http://arxiv.org/abs/1412.6980 (2014).

[52] Ni J, Wu J, Tong J, Chen Z, Zhao J (2019). GC-Net: Global context network for medical image segmentation. Comput Methods Prog Biomed.

[53] Azzopardi G, Strisciuglio N, Vento M, Petkov N (2015). Trainable COSFIRE filters for vessel delineation with application to retinal images. Med Image Anal.

[54] Roychowdhury S, Koozekanani DD, Parhi KK (2015). Blood vessel segmentation of fundus images by major vessel extraction and subimage classification. IEEE J Biomed Health Inform.

[55] Zhao Y, Rada L, Chen K, Harding SP, Zheng Y (2015). Automated vessel segmentation using infinite perimeter active contour model with hybrid region information with application to retinal images. IEEE Trans Med Imaging.

[56] Fu H, Xu Y, Lin S, Wong DWK, Liu J. Deepvessel: retinal vessel segmentation via deep learning and conditional random field. In: International conference on medical image computing and computer-assisted intervention. 2016.

[57] Li Q, Feng B, Xie LinPei, Liang P, Zhang H, Wang T (2015). A cross-modality learning approach for vessel segmentation in retinal images. IEEE Trans Med Imaging.

[58] Melinščak M, Prentašić P, Lončarić S. Retinal vessel segmentation using deep neural networks. In: 10th International conference on computer vision theory and applications (VISAPP 2015). 2015.

[59] He K, Girshick R, Dollár P. Rethinking imagenet pre-training. arXiv preprint http://arxiv.org/abs/1811.08883 (2018).

